# A Comparative Study of Cytotoxicity of PPG and PEG Surface-Modified 2-D Ti_3_C_2_ MXene Flakes on Human Cancer Cells and Their Photothermal Response

**DOI:** 10.3390/ma14164370

**Published:** 2021-08-04

**Authors:** Bushra Rashid, Ayaz Anwar, Syed Shahabuddin, Gokula Mohan, Rahman Saidur, Navid Aslfattahi, Nanthini Sridewi

**Affiliations:** 1 Faculty of Defence Science and Technology, National Defence University of Malaysia, Kuala Lumpur 57000, Malaysia; whiterose4626@gmail.com; 2 Primary & Secondary Health Care Department, Govt. of Punjab, Lahore 54000, Pakistan; 3 Department of Biological Sciences, School of Medical and Life Sciences, Sunway University, Subang Jaya 47500, Malaysia; 4 Department of Chemistry, School of Technology, Pandit Deendayal Energy University, Gandhinagar 382007, India; 5 Faculty of Applied Sciences, Universiti Teknologi MARA, Cawangan Negeri Sembilan, Kampus Kuala Pilah, Kuala Pilah, Shah Alam 40450, Malaysia; 6 Institute of Biological Sciences, Faculty of Science, University of Malaya, Kuala Lumpur 50603, Malaysia; g.mohan@um.edu.my; 7 Research Centre for Nano-Materials and Energy Technology (RCNMET), School of Engineering and Technology, Sunway University, Petaling Jaya 47500, Malaysia; saidur@sunway.edu.my; 8 Department of Mechanical Engineering, Faculty of Engineering, University of Malaya, Kuala Lumpur 50603, Malaysia; navid.fth87@yahoo.com

**Keywords:** MXene, anticancer, PPG, PEG, PTT, cytotoxicity

## Abstract

The MXenes are a novel family of 2-D materials with promising biomedical activity, however, their anticancer potential is still largely unexplored. In this study, a comparative cytotoxicity investigation of Ti_3_C_2_ MXenes with polypropylene glycol (PPG), and polyethylene glycol (PEG) surface-modified 2-D Ti_3_C_2_ MXene flakes has been conducted towards normal and cancerous human cell lines. The wet chemical etching method was used to synthesize MXene followed by a simple chemical mixing method for surface modification of Ti_3_C_2_ MXene with PPG and PEG molecules. SEM and XRD analyses were performed to examine surface morphology and elemental composition, respectively. FTIR and UV-vis spectroscopy were used to confirm surface modification and light absorption, respectively. The cell lines used to study the cytotoxicity of MXene and surface-modified MXenes in this study were normal (HaCaT and MCF-10A) and cancerous (MCF-7 and A375) cells. These cell lines were also used as controls (without exposure to study material and irradiation) to measure their baseline cell viability under the same lab environment. The surface-modified MXenes exhibited a sharp reduction in cell viability towards both normal (HaCaT and MCF-10A) and cancerous (MCF-7 and A375) cells but cytotoxicity was more pronounced towards cancerous cell lines. This may be due to the difference in cell metabolism and the occurrence of high pre-existing levels of reactive oxygen species (ROS) within cancerous cells. The highest toxicity towards both normal and cancerous cell lines was observed with PEGylated MXenes followed by PPGylated and bare MXenes. The normal cell’s viability was barely above 70% threshold with 250 mg/L PEGylated MXene concentration whereas PPGylated and bare MXene were less toxic towards normal cells, even at 500 mg/L concentration. Moreover, the toxicity was found to be directly related to the type of cell lines. In general, the HaCaT cell line exhibited the lowest toxicity while toxicity was highest in the case of the A375 cell line. The photothermal studies revealed high photo response for PEGylated MXene followed by PPGylated and bare MXenes. However, the PPGylated MXene’s lower cytotoxicity towards normal cells while comparable toxicity towards malignant cells as compared to PEGylated MXenes makes the former a relatively safe and effective anticancer agent.

## 1. Introduction

Cancer is the major cause of death among non-communicable diseases and acts as a barrier to increasing life expectancy in the 21st century. The World Health Organization (WHO) report 2015, revealed that cancer is the first or second leading cause of mortality before 70 years of age in 91 of 172 countries worldwide [1,2].

For the treatment of cancers, various approaches such as surgery, chemotherapy, radiotherapy, immunotherapy, and hormone therapy are in use. Among these, chemotherapy and radiotherapy therapy have been widely used to treat most cancer types. These treatment modalities have deleterious side effects due to high energy irradiation exposure and cytotoxic drugs, which are toxic to normal tissues as well. Furthermore, these techniques do not guarantee a satisfying cure.

Recently, low energy photothermal therapy (PTT) to fight against cancer has gained immense scientific interest due to its utilization of low energy radiations (visible to the infrared range) and effective hyperthermic effect. The principle of PTT is the production of heat energy near tumor cells. The heating effect would successfully kill the cancer cells without harming the normal cells as cancerous cells are more sensitive to high temperatures as compared to healthy cells [3]. The ideal PTT agent should be photoresponsive and harmless for healthy cells, and the affected areas can be selectively targeted through laser irradiation [4].

With the advancement in nanotechnology, various nanoparticles, i.e., gold (Au) nanoparticles and surface-modified Au particles [5], silver (Ag) [6], carbon nanotubes [3], graphene [3], molybdenum oxide (MoOx) [7], tungsten disulfide (WS_2_) [3], copper selenide (CuSe) [8], self-assembled organic polyamic materials [9], and dyes have been successfully demonstrated to have promising cytotoxicity towards normal and cancerous cells and high photoresponse in the biocompatible infrared region. Other than the photothermal effect, the synergistic effect of PTT agents as drug carriers has also been investigated with reasonable success [10].

Recently, a new class of materials, two-dimensional (2-D) transition metal carbides/nitrides (MXenes), has been widely investigated in the biomedical field due to their biocompatibility, wide surface area, and high photoresponse. The MXenes (“M_n+1_X_n_T_x_”), X is for nitrogen and/or carbon, M is any ‘transition metal”, and T can be any surface functional group. The 2-D sheet-like structures are obtained from selective etching of the A-group element from its most common precursor in MAX phase ceramic material by using strong acid-like hydrofluoric acid [11]. In a study, the cytotoxicity of MXenes (Ti_3_C_2_) were investigated against normal and cancerous cells. The results revealed high cytotoxicity towards cancerous cells and a lower effect on normal cells under room temperature without external stimulation [12]. In another study, Ti3C_2_ was surface modified with doxorubicin (DOX) for enhanced effectiveness and photoactive drug release on the target area [13]. In a further study, Ti_3_C_2_ modified with soybean phospholipids [14] and polyethylene glycol (PEG) also showed promising results as anticancer agents.

Although the MXenes showed promising results as anticancer agents and are relatively less toxic to normal cells, there is still very scant literature available and further studies need to be conducted. Moreover, the PEGylated MXenes showed an increase in cytotoxicity towards normal cells as compared to bare MXenes. After 24 h of exposure with high concentrations of PEG MXenes, the normal cells, i.e., HaCaT and MCF-10 showed cell viability around 70%, which is comparable to the cancer cell line, A375. After 48 h of exposure to high concentrations of PEGylated MXenes, the cell viability of healthy cells also dropped below 60% [15]. Moreover, depending on the manufacturing process, PEGs may include detectable quantities of ethylene oxide and 1,4-dioxane [16]. The International Agency for Research on Cancer classifies ethylene oxide as a known human carcinogen, while 1,4-dioxane is listed as a possible human carcinogen [17]. Ethylene oxide has also been categorized as a developmental toxicant by the California Environmental Protection Agency, based on findings that it may interfere with human development [18]. Therefore, an attempt should be made to find relatively safer MXenes via different surface modifications.

In this study, for the first time, the synthesis and the cytotoxicity potential of polypropylene glycol (PPG) surface-modified Ti_3_C_2_ MXene against normal and malignant cancer cell lines has been investigated. The cytotoxicity of PPGylated MXene was compared with PEGylated MXene and bare MXenes to find the anticancer agent with the least toxicity towards healthy cells. The photothermal effect of modified MXenes was also measured and compared with bare MXene using 808 nm laser light.

The null hypotheses of the present study were: (1) there is no significant difference in the cytotoxic potential of bare Ti_3_C_2_ MXene, Ti_3_C_2_-PEGylated MXene, and Ti_3_C_2_-PPGylated MXene on human cancer cells and (2) there is no significant difference in the photothermal therapy potential of bare Ti_3_C_2_ MXene, Ti_3_C_2_-PEGylated MXene, and Ti_3_C_2_-PPGylated MXene against human cancer cells.

## 2. Materials and Method

### 2.1. MXene Synthesis

2-D Ti_3_C_2_ MXene has been synthesized through selective etching of MAX phase (Ti_3_AlC_2_) as described in our previous work [19]. In brief, a 2 molar solution of ammonium hydrogen difluoride (NH_4_HF) was prepared in DI water by stirring for 1 h at room temperature. Afterward, the etching process was followed by the addition of 1g of Ti_3_AlC_2_ material to the homogenous solution. The addition of Ti_3_AlC_2_ to the solution was conducted slowly to avoid overheating (exothermic reaction), followed by stirring at 40 °C for 48 h. After completion of the etching process, a dilute solution of NaOH was added slowly until the pH of the solution reached 6, followed by filtering and rinsing the solid product using DI water several times. The product was washed further 4 times at 3500 rpm using an ultrahigh-speed centrifuge. Then the achieved multi-layered Ti_3_C_2_T_x_ was dispersed in isopropyl alcohol and sonication was done for one hour for delamination. To get delaminated MXene, the power of the sonicator was set to 70%, and the switching time was set to 7/3 s. Then the obtained MXene flakes were dried for 12 h using a vacuum oven. A stock of 15 g MXene in powder form was prepared and stored in the dark condition below 4 °C for future use.

### 2.2. Surface Modificationof Ti_3_C_2_ MXene with PPG and PEG

For the surface functionalization of Ti_3_C_2_ MXene, 5 g MXene was mixed with 100 mL of PPG and PEG respectively, sonicated for 1 h in two intervals, and stirred overnight using a magnetic stirrer. The stock solution was then treated with DI water and functionalized particulates were separated from the liquid phase using a centrifuge (6000 rpm). The functionalized particulates were rinsed with DI water five times and stored in glass vials under dark conditions. A defined amount of functionalized and bare particulates was dispersed in DI water before use.

### 2.3. Characterization

Scanning electron microscopy (SEM) images were obtained using a Mira3–SAMX TESCAN (Kohoutovice, Brno, Czech Republic) in secondary electron mode to examine the surface morphology of as-synthesized MXene. X-ray diffraction (XRD) patterns were recorded using a Bruker D8-Discover machine (Bruker Co., Karlsruhe, Germany) to confirm the composition of MXene and the proper etching of Al from the MAXphase. The IRAffinity-1S Fourier Transformed Infrared (FTIR) spectrometer (Shimadzu, Kyoto, Japan) was utilized to confirm surface modification of MXene with PEG and PPG.

### 2.4. In Vitro Cytotoxicity Assays

The effect of 2-D Ti_3_C_2_ MXene, Ti_3_C_2_-PEG MXene, and Ti_3_C_2_-PPG MXene on human cell lines was studied in a lab environment by gradually increasing the concentrations of the concerned material from 0 mg/L to 500 mg/L. The cell lines included in the study were MCF-7 (human breast cancer cells; ATCC, Manassas, VA, USA), MCF-10A (normal human mammary epithelial cells; Thermo Fisher Scientific, Waltham, MA, USA), A375 (human skin malignant melanoma cells; ATCC, Manassas, VA, USA), and HaCaT (human immortalized keratinocytes; Thermo Fisher Scientific, Waltham, MA, USA). DMEM (Sigma-Aldrich, St. Louis, MO, USA) culture medium was used for MCF-7, A375, and HaCaT cell lines, whereby this culture medium was supplemented with fetal bovine serum (FBS) with a strength of ten percent volume/volume. The necessary antibiotics were also added, which were one percent of streptomycin and penicillin (*v/v*) respectively. An essential amino acid, one percent of L-glutamine (*v/v*), was also added. While for the culture of MCF-10A cells, DMEM medium was supplemented with 5% (*v/v*) horse serum, human insulin (10 µg/mL), the epithelial growth factor (10 ng/mL), and hydrocortisone in the strength of 5 μg/mL. Other culture requirements were 5% CO_2_, 95% humidity, and 37 °C temperature.

The methyl tetrazolium (MTT) assay was used to measure in vitro cytotoxicity of the surface-modified MXenes. Each of the four cell lines was inoculated in a 96 well plate with each well containing 1 × 10^4^ cells. Then, incubation was carried out to ensure surface adherence of the cells. After removal of the supernatant, the bare MXene, PEGylated MXene, and PPGylated MXenes in different concentrations were added separately to the well plates, followed by incubation for 24 h. The control cell lines were cultured and incubated with fresh growth medium without MXenes. After the completion of incubation time with studied materials, phosphate-buffered saline (PBS, Sigma-Aldrich) was used to wash the cells. Then, for each well, 100 μL of MTT (Sigma-Aldrich) solution was added in a concentration of 0.5 mg/mL in PBS. The cell lines were kept away from light. Incubation with MTT solution was performed for 4 h. After careful removal of the supernatant, 100 μL/well of Dimethyl Sulfoxide (DMSO, Sigma-Aldrich) solution was added to dissolve the “violet formazan crystals” and spectrophotometric absorbance was determined at 570 nm. By using Equation (1), the cell viability was calculated in percentage in comparison to control cell lines.

(1) $$\mathrm{Cellular}\;\mathrm{viability}=\;\frac{a_{i}}{a_{c}}\;\times 100\%$$

a_i_ = the average of spectrophotometric absorbance for the studied material group,
a_c_ = the average of spectrophotometric absorbance for the control group.

Cytotoxicity tests for each cell line exposed to MXene, PEGylated MXene, and PPGylated MXene samples, with and without irradiation, were conducted using a set of five replicates for each concentration and expressed as mean ± standard error.

### 2.5. Photothermal Testing

**Figure 1:**
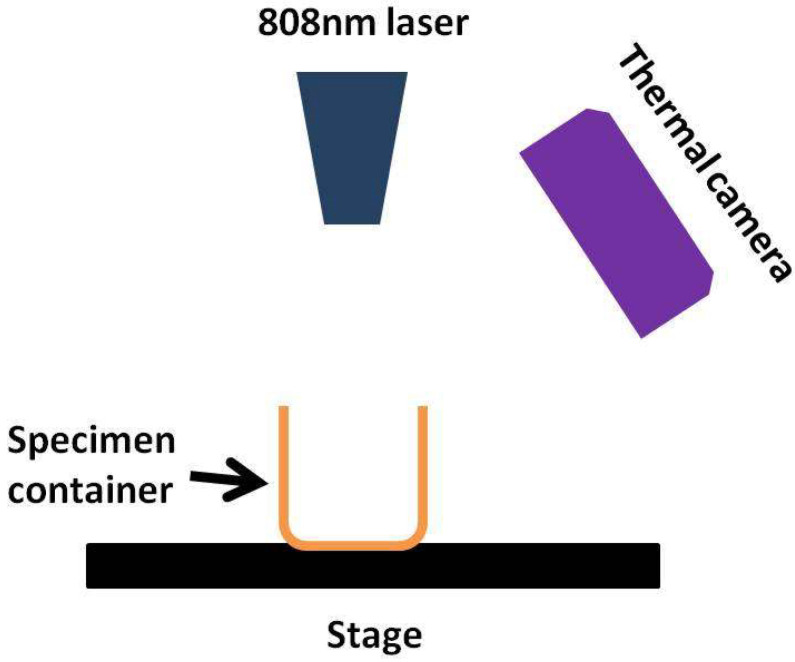
Schematic arrangement for photothermal testing.

A test apparatus was developed to irradiate the specimens with infrared radiation (IR) using a Niahode IR Laser Diode (China). Figure 1 shows the schematic illustration of the photothermal apparatus. A focusable “Niahode” 808 nm laser diode was employed along with a thermal imaging camera (Cat S60, Bullitt Group Ltd., Berkshire, UK) to measure an increase in temperature. The power of the laser was set at 500 mW/cm^2^. The bare and surface-modified MXenes (0.2 mg) were each suspended in DI water (10 mL) in a specimen container, and irradiated for 5 min prior to cytotoxicity analysis. 

### 2.6. Statistical Analysis

Statistical analysis was performed using GraphPad Prism 9.2.0 (GraphPad Software, San Diego, CA, USA). Data resulting from cytotoxicity experiments were subjected to one-way analysis of variance (ANOVA) followed by Tukey’s post hoc test for comparison between test groups of every tested concentration. Significance level was set at *p* < 0.05.

## 3. Results and Discussion

**Figure 2:**
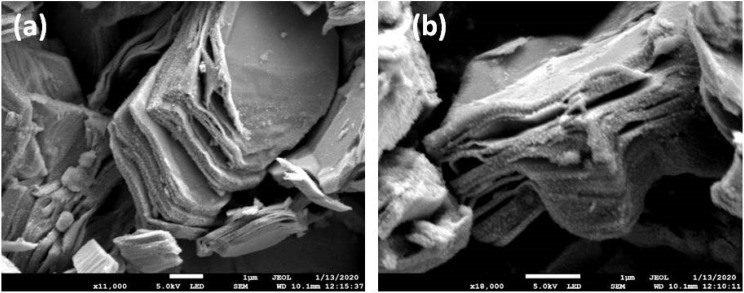
SEM micrographs of as-synthesized MXene at (**a**) 11,000× and (**b**) 18,000× magnification.

Figure 2a,b show the SEM micrographs of as-synthesized MXenes. The delamination and layered structure of MXenes is evident from the SEM images. The thickness of sheet-like MXene is approximately 1 nm whereas other dimensions are in the micrometer range. The proper sonication of nano-particulates leads to proper delamination. Furthermore, the surface of MXene nano-sheets is smooth without any dips and/or excessive roughness.

**Figure 3:**
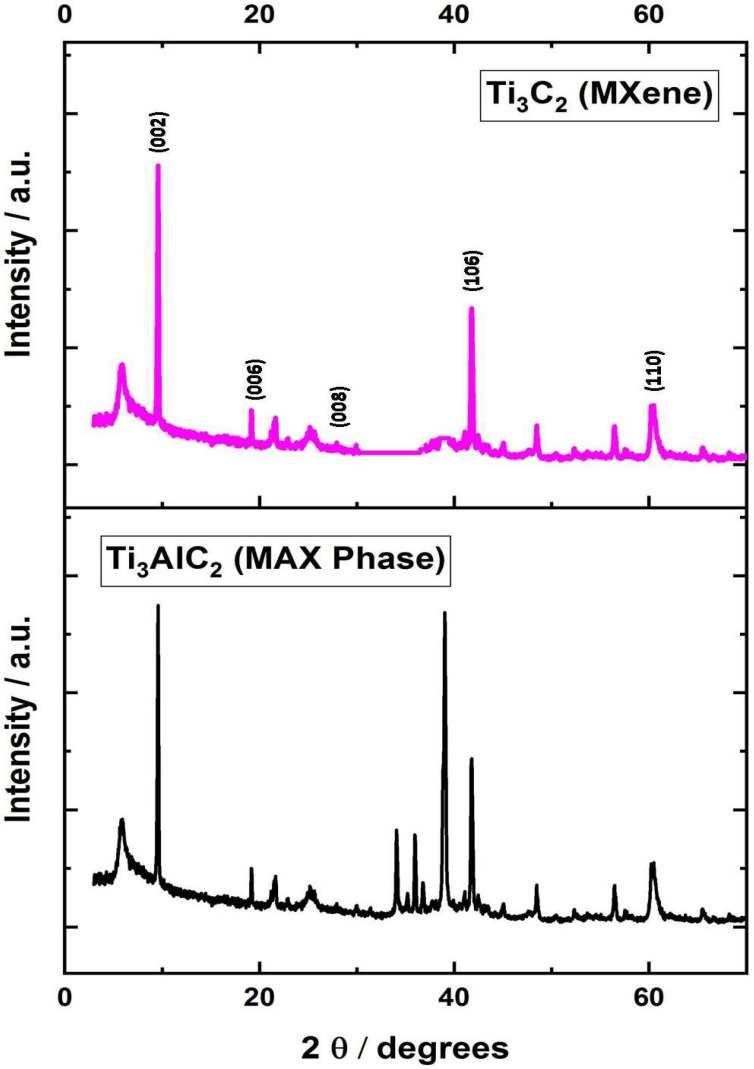
XRD pattern of as-synthesized MXene.

The XRD analysis was performed to verify the formation of MXene and proper etching of Al from the MAX phase. Figure 3 shows the X-ray pattern of as-synthesized and washed MXene powder along with the precursor, i.e., MAX Phase. The indices mentioned in Figure 3, i.e., (002), (006), (008), (106), and (110) represent MXene structure [20]. The most intense peak at 2θ: 38 degrees (JCPDS No: 52-0875) [21] that corresponds to Al disappeared, which confirmed the proper etching of Al from the MAX phase.

**Figure 4:**
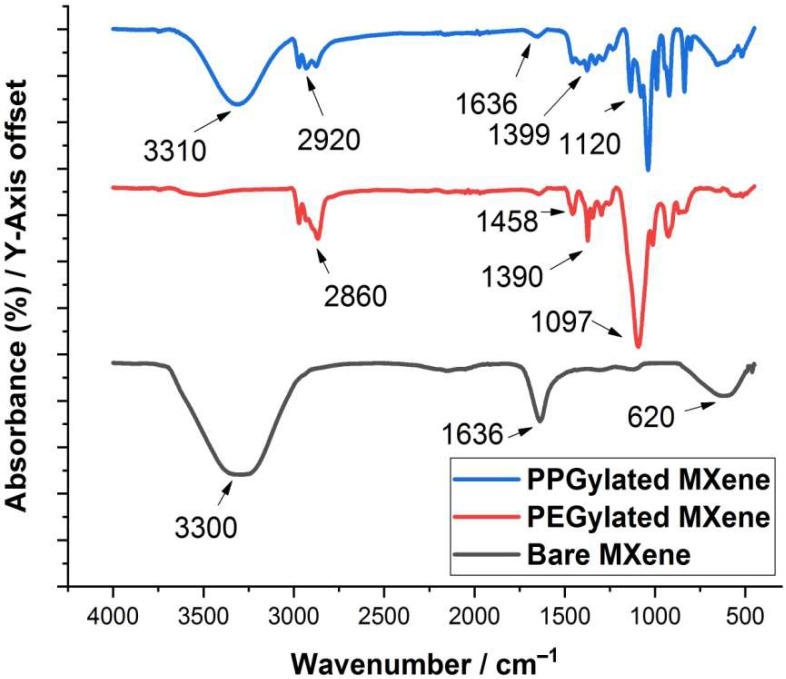
Comparison of FTIR spectra of bare Mxene and surface-modified MXenes.

Figure 4 represents the FTIR spectra of surface-modified and bare MXenes. As apparent from the observed data, the broad absorption peaks are observed at 1636 cm^−1^ and 3310 cm^−1^, which confirms the presence of the hydroxyl group. In all of the specimens, these were assigned to the absorbed moisture from the environment and highly hydrogen-bonded OH or extremely strong coordinated H_2_O. Furthermore, a peak at 620 cm^−1^ was possibly due to the Ti-O bond deformation vibration [22]. As evident from the FTIR results, the characteristic C-O stretching (1000–1200 cm^−1^), similar to alcohol and ethers, C-H bending (1400–1500 cm^−1^), and C-H stretching (2850–3000 cm^−1^) peaks can be observed in the case of PEGylated and PPGylated MXenes. The C-O stretching is seen at 1120 cm^−1^ for PEG and 1097 cm^−1^ for PPG. The aliphatic C-H stretching peak is observed at 2901 cm^−1^ for PEG and at 2860 cm^−1^ for PPG. The C-H bending vibration is seen at 1399 cm^−1^ and 1120 cm^−1^ for PEG and 1458 cm^−1^ and 1390 cm^−1^ for PPG. These findings confirm that the surface modification of MXene with PEG and PPG were successfully achieved [23,24].

**Figure 5:**
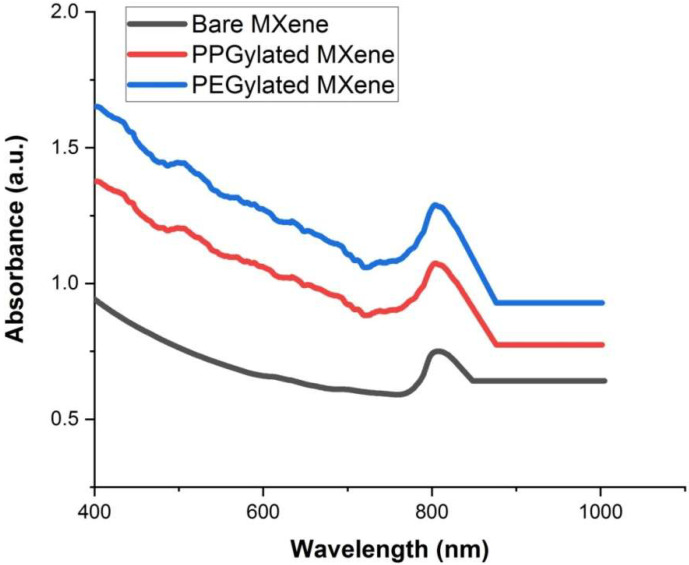
UV-Vis spectra of bare Mxene and surface-modified MXenes.

To identify the light absorption properties, UV-vis spectroscopy of bare MXene and functionalized MXenes were performed. Figure 5 shows the UV-vis spectra of specimens under investigation. A sample concentration of 0.2 mg/L of was prepared in DI water for the UV-vis spectroscopy. The absorption peak of the bare MXene was observed in the near-infrared region, which is in line with the findings in literature [25]. The absorption peak of PEGylated and PPGylated MXenes were in the same region of the electromagnetic spectrum. It can be observed that the same concentration of functionalized MXenes exhibited higher absorption compared to bare MXene. The highest absorption was found for PEGylated MXenes followed by PPGylated MXene and bare MXene.

**Figure 6:**
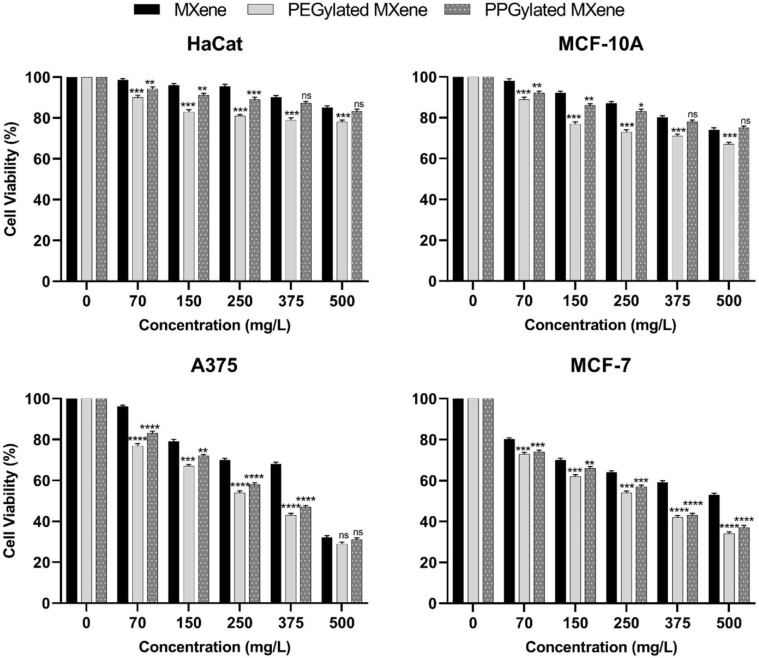
In vitro cytotoxicity against normal (HaCaT & MCF-10A) and cancer (MCF-7 & A375) cell lines after 24 h of exposure to increasing concentrations of MXene, PEGylated MXene, and PPGylated MXene. Cytotoxicity results are expressed as mean ± standard error. Statistical analysis was performed using one-way ANOVA, followed by Tukey’s post hoc test, where * *p* < 0.05, ** *p* < 0.01, *** *p* < 0.001, and **** *p* < 0.0001 were considered statistically significant.

Figure 6 shows the percent change in cell viability with respect to the concentration of reference bare MXene, PEGylated MXene, and PPGylated MXene, respectively. The selected incubation time was 24 h as mentioned in the literature [13]. Various other studies used different time selections for cytotoxicity assessment [26]. A statistically significant decrease in cell viability was observed across all tested cell types exposed to PEG and PPG modified MXenes as compared to bare MXene (*p* < 0.05) except for normal cell lines (HaCaT and MCF-10A) at 375 mg/L and 500 mg/L where the cytoxicity caused by bare MXene and PPGylated MXene were comparable. However, all of the test samples were relatively more toxic towards malignant cells as compared to normal cells. This can be explained on the basis of the differences in cellular metabolism and permeability. MXene exerts its biological activity on living cells by the generation of reactive oxygen species (ROS). This property makes MXenes exhibit anticancer potential due to the already increased baseline amount of reactive oxygen species within cancer cells, owing to increased anabolic and catabolic reactions [27]. ROS plays a key role in cell metabolism and survival, as well as in the cytotoxicity mechanisms of various classes of carbon-based nanomaterials [12].

It was observed that the MXene cytotoxicity was also directly related to the type of cell lines, the minimum for HaCaT cell lines and the maximum towards A375 cell lines. A sharp increase in toxicity towards both the normal and cancerous cell lines was observed for PEGylated and PPGylated MXenes. At higher concentrations, the normal cell’s viability dropped to below 70% for PEGylated MXenes, thus, according to ISO standard (ISO 10993-1:2018) [28], the growth of normal cells was also restricted. In the case of PPGylated MXene, the reduction in viability of normal cells was observed but not as strong as with PEGylated MXene. The viability of normal cells was observed to be higher than 70%, even at the highest concentration of PPGylated MXenes. The viability below 70% for cancerous cell lines was observed at 375 mg/L in bare MXenes and 150mg/L for PEGylated and PPGylated MXenes. At 150 mg/L of PEGylated MXene, the viability of both cancer cell lines (MCF-7 and A375) dropped below 70%, while for PPGylated MXene at 150 mg/L, only the viability of MCF-7 cell line dropped below 70%. The A375 cell line, however, needed a higher concentration of PPGylated MXene to produce a similar effect. This may be due to the bulky pendant-like structure of PPG that reduces the cell wall penetration compared to PEG. While PEG has a hydrophilic nature that causes it to be used widely as a surface penetration enhancer [29]. At concentrations above 250 mg/L, the drop in cell viability of MCF-7 was comparable for both PEGylated and PPGylated MXenes. There was no significant difference between these two surface modified MXenes at 375 mg/L (*p* = 0.7046) and 500 mg/L (*p* = 0.05). Interestingly, at 500 mg/L sample concentration, both bare and surface-modified MXenes showed no significant difference in the cytotoxicity towards A375 cells (*p* > 0.05). On the other hand, for normal cell lines, the cell viability was well above 70% when exposed to bare MXene and PPGylated MXene even at 500 mg/L concentration, while with PEGylated MXene, the MCF-10A viability started to drop from 250 mg/L onwards. The HaCaT cell line was the least affected of all the studied samples, which might be because of its immortalized nature.

**Figure 7:**
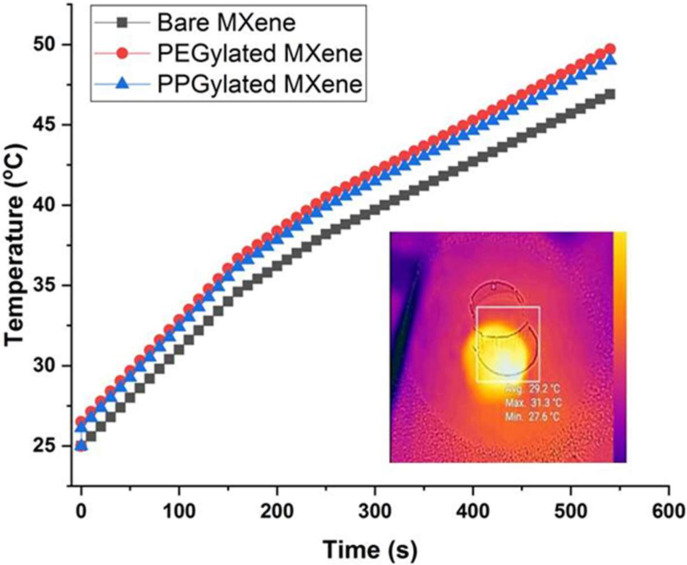
Photothermal response of bare MXene and surface-modified MXenes.

The relationship between temperature and irradiation exposure time is presented in Figure 7. A laser diode of 808 nm irradiation was employed at 500 mW/cm^2^ intensity. A steady increase in temperature of samples was observed by irradiation with near-infrared wavelength. The temperature response was not largely affected by surface modification.

The highest increase in the rate of temperature was observed in the case of PEGylated MXene followed by PPGylated MXene and bare MXene. This increase can be explained based on the material’s suspension stability under irradiation conditions, which resulted in improved efficiency of the conversion of light to thermal energy [15]. Moreover, the surface-modified MXenes exhibited more light absorption, as can be seen in Figure 5. This effect leads to increased light energy absorption and activation of MXenes to an excited state where it then releases vibrational energy (heat), which raises the temperature. Thus, PEGylated MXenes had the highest energy absorption spectra, and thus, higher heat energy and higher temperature, followed by PPGylated MXenes and bare MXenes.

**Figure 8:**
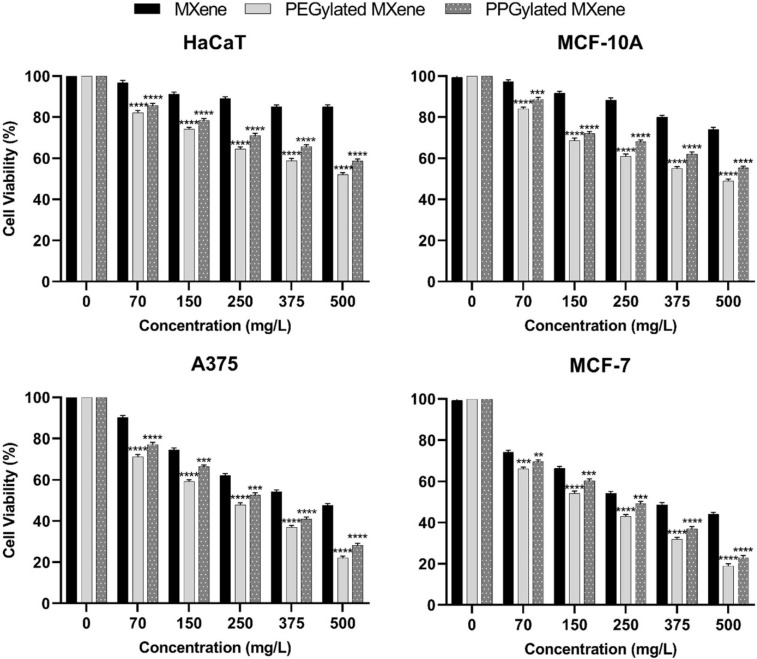
In vitro cytotoxicity against normal (HaCaT & MCF-10A) and cancer (MCF-7 & A375) cell lines after 24 h incubation with increasing concentrations of infrared irradiated MXene, PEGylated MXene, and PPGylated MXene. Cytotoxicity results are expressed as mean ± standard error. Statistical analysis was performed using one-way ANOVA, followed by Tukey’s post hoc test, where * *p* < 0.05, ** *p* < 0.01, *** *p* < 0.001 and **** *p* < 0.0001 were considered statistically significant.

The percentage change in cell viability after incubation with increasing concentration of irradiated bare MXene, PEGylated MXene, and PPGylated MXene was also observed and is presented in Figure 8. The irradiation time was set to 5 min at 500 mW/cm^2^ irradiation intensity.

The rise in sample concentration led to a reduction in the cell viability of all tested cell lines. The cytotoxicity effect of surface-modified MXenes were significantly higher than the bare MXene at all concentrations regardless of the cell type. Furthermore, the cell viability of cancerous cells (i.e., MCF-7 and A375) dropped to 70% at just 70 mg/L concentration of PEGylated MXene. In the case of PPGylated MXene, the cell viability of only MCF-7 cancerous cells dropped to below 70% at 70 mg/L while the A375 cell line required 150 mg/L of PPGylated MXene to achieve below 70% cell viability. As for bare MXene, the concentration required to induce a similar effect in the A375 cell line was 250 mg/L. At higher sample concentrations and the same irradiation level, the normal cells were also affected. At 150 mg/L concentration of PEGylated MXene, the cell viability of normal cells was barely above 70% whereas, in the case of PPGylated MXene, a similar effect was only observed at 250 mg/L. Generally, the irradiated surface-modified MXenes showed a marked increase in the toxicity against cancer cell lines as compared to similar MXene samples without irradiation.

**Figure 9:**
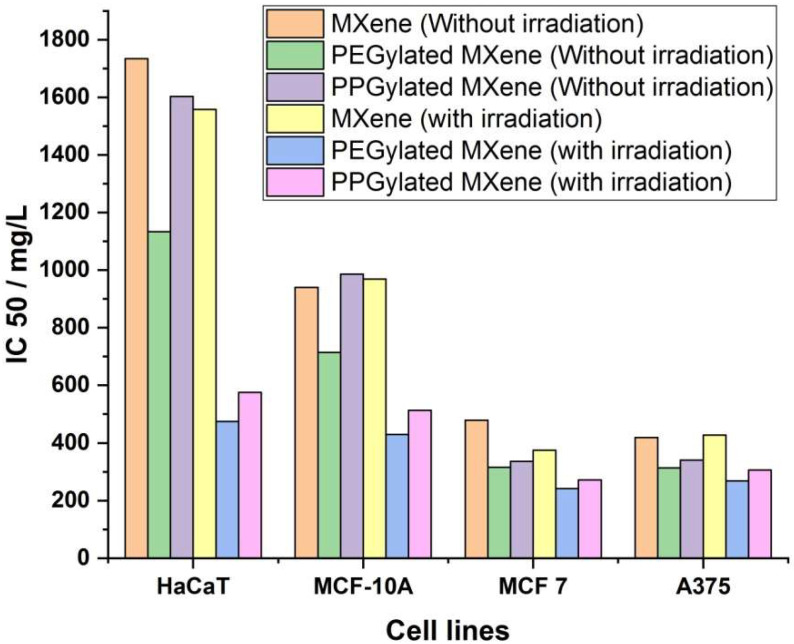
IC50 values of specimens under investigation with and without irradiation.

The half-maximal inhibitory concentration (IC50) was also calculated using the linear extrapolation method (using ED50 plus v 1.0 software) and presented in Figure 9. The IC50 value of bare MXene in the case of normal cell lines, i.e., HaCaT and MCF-10A, without irradiation was calculated to be 1734.77 mg/L and 940.43 mg/L, respectively, whereas, for malignant cell lines, i.e., MCF-7 and A375, the IC50 values were 478.69 mg/L and 419.18 mg/L, respectively. This again confirms that MXenes are more toxic towards malignant cells. A low IC50 values for PEGylated MXene towards normal cell lines, i.e., HaCaT (1133.38 mg/L) and MCF-10A (714.40 mg/L), was observed even without irradiation.

On the one hand, in the case of PPGylated MXene, the IC50 values for HaCaT and MCF-10A were calculated to be 1602.80 mg/L and 985.95 mg/L, respectively, which are much closer to bare MXene. The IC50 values of PEGylated and PPGylated MXenes for malignant cell lines were comparable, where the values for MCF-7 were 315.81 mg/L and 313.51 mg/L respectively while for A375, the IC50 values were 335.93 mg/L and 340.12 mg/L respectively, without irradiation. A further reduction of IC50 values towards both normal and malignant cells was observed when the samples were irradiated using an 808 nm laser at a low power of 500 mW/cm^2^. IC50 values after irradiation for bare MXene were 1597.96 mg/L for HaCaT, 968.67 mg/L for MCF-10A, 374.49 for MCF-7, and 427.54 mg/L for A375. IC50 values after irradiation for PEGylated MXene were 474.66 mg/L for HaCaT, 429.83 mg/L for MCF-10A, 241.16 mg/L for MCF-7, and 268.83 mg/L for A375. The IC50 values after irradiation for PPGylated MXene were 576.03 mg/L for HaCaT, 513.24 mg/L for MCF-10A, 272.04 mg/L for MCF-7, and 305.87 mg/L for A375. The cytotoxicity effect on malignant cells was more pronounced. It can be observed that the PEGylated MXene were the most toxic towards malignant cell lines followed by the PPGylated MXenes and bare MXenes. On the other hand, bare MXene is the least toxic towards normal cell lines followed by PPGylated MXene and PEGylated MXene.

**Figure 10:**
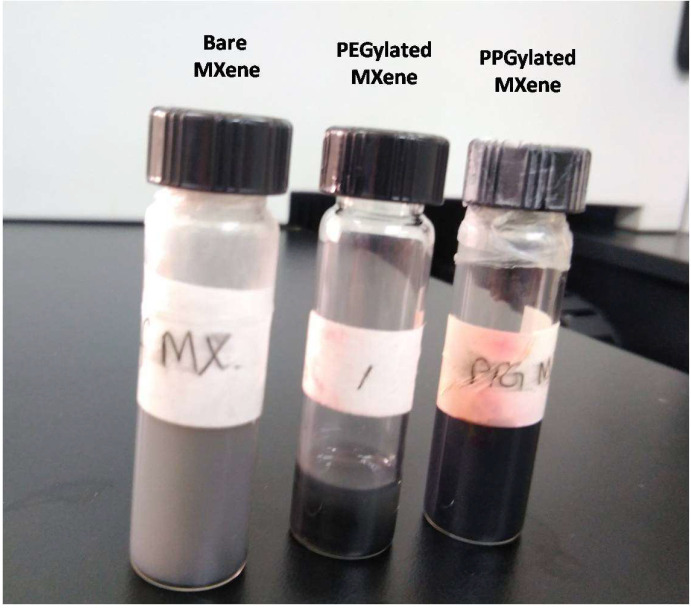
The suspension was kept for 1 week in dark conditions at room temperature.

The shelf life observation of bare and surface-modified MXenes was also observed. Figure 10 shows that the color of bare MXene changed after being kept in 1 week in dark condition at room temperature (25–28 °C). On the other hand, no visible changes in modified MXenes suspended in DI water were observed after 1 week under similar conditions. This observation is in line with those reported in literature [30,31].

## 4. Conclusions

In this study, a comparative cytotoxicity investigation has been conducted for PEGylated and PPGylated MXene nanoflakes/sheets against normal cell lines and cancerous cell lines. The toxicity is strongly related to the type of cell lines and a more pronounced toxicity effect has been observed for cancerous cell lines compared to normal cell lines (with and without irradiation). Further, it has been observed that PEGylated MXenes are more toxic towards cancerous cell lines followed by PPGylated and bare MXenes. The effect becomes more pronounced at higher concentrations and with irradiation. Furthermore, it was found that surface modification of MXene with PPG proves relatively safer to normal cell lines as compared to PEG based MXene, even at high concentrations and also with irradiation. Without irradiation, PPGylated MXene caused similar toxicity to MCF-7 cell line (cancerous cell line) as PEG-modified MXene but lower toxicity for A375 cell lines. However, after irradiation, PPG and PEG-modified MXenes had comparable IC50 values for both MCF-7 and A375 malignant cell lines. These results suggest that PPG modified MXene may prove to be a safer photothermal and anticancer agent as compared to PEG-modified MXene and thus, can be further explored in cancer therapies. Moreover, systematic in vivo studies using animal models should be performed to further investigate the anticancer potential and possible side effects of the PPG surface modified MXene.

## Data Availability

The datasets used and/or analyzed during the current study are available from the corresponding author on reasonable request.

## Abbreviations

WHO	World Health Organization
PTT	photothermal therapy
Au	Gold
Ag	Silver
MoOx	molybdenum oxide
WS2	tungsten disulfide
CuSe	copper selenide
2-D	two-dimensional
MXenes	transition metal carbides/nitrides
DOX	Doxorubicin
PEG	polyethylene glycol
PPG	polypropylene glycol
SEM	Scanning electron microscopy
XRD	X-ray diffraction
FTIR	Fourier transform infrared
MCF-7	human breast cancer cells
MCF-10A	normal human mammary epithelial cells
A375	human skin malignant melanoma cells
HaCaT	human immortalized Keratinocytes
FBS	fetal bovine serum
MTT	methyl tetrazolium
PBS	phosphate-buffered saline
DMSO	dimethyl sulfoxide
IR	infrared radiation
